# Obstetrician-Gynecologists' Strategies for Patient Initiation and Maintenance of Antiobesity Treatment with Glucagon-Like Peptide-1 Receptor Agonists

**DOI:** 10.1089/jwh.2020.8683

**Published:** 2021-07-12

**Authors:** Lisa Gill, Suzanne Mackey

**Affiliations:** ^1^ Department of Obstetrics, Gynecology and Women's Health, University of Minnesota, Minneapolis, Minnesota, USA.; ^2^ Salvéo Weight Management, Voorhees Township, New Jersey, USA.

**Keywords:** obesity, weight loss, GLP-1 RA, treatment initiation, treatment adherence

## Abstract

Obesity is a chronic disease affecting women at higher rates than men. In an obstetrics and gynecology setting, frequently encountered obesity-related complications are polycystic ovary syndrome, fertility and pregnancy complications, and increased risk of breast and gynecological cancers. Obstetrician-gynecologists (OBGYNs) are uniquely positioned to diagnose and treat obesity, given their role in women's primary health care and the increasing prevalence of obesity-related fertility and pregnancy complications. The metabolic processes of bodyweight regulation are complex, which makes weight-loss maintenance challenging, despite dietary modifications and exercise. Antiobesity medications (AOMs) can facilitate weight loss by targeting appetite regulation. There are four AOMs currently approved for long-term use in the United States, of which liraglutide 3.0 mg is among the most efficacious. Liraglutide 3.0 mg, a glucagon-like peptide-1 receptor agonist (GLP-1 RA), is superior to placebo in achieving weight loss and improving cardiometabolic profile, in both clinical trial and real-world settings. In addition, women with fertility complications receiving liraglutide 1.8–3.0 mg can benefit from improved ovarian function and fertility. Liraglutide 3.0 mg is generally well tolerated, but associated with transient gastrointestinal side effects, which can be mitigated. In this review, we present the risks of obesity and benefits of weight loss for women, and summarize clinical development of GLP-1 RAs for weight management. Finally, we provide practical advice and recommendations for OBGYNs to open the discussion about bodyweight with their patients, initiate lifestyle modification and GLP-1 RA treatment, and help them persist with these interventions to achieve optimal weight loss with associated health benefits.

## Introduction

Obesity, defined as body mass index (BMI) ≥30, affects around 42% of adults in the United States.^1^ While the overall prevalence of obesity in years 2017/2018 was similar between women and men (42.1% and 43.0%, respectively), women had a higher prevalence of severe obesity (class III, BMI ≥40 kg/m^2^) than men (11.5% vs. 6.9%, respectively).^1^ The prevalence of obesity among adolescent girls (12–19 years) in 2015/2016 was 20.9%.^2^ The chronic abnormal fat accumulation that characterizes obesity^3^ has far-reaching effects on women's health.^4^ Adipose tissue is not simply a storage of fat; it acts as an endocrine organ involved in many biological functions, including reproduction.^5^

In women, obesity is associated with polycystic ovary syndrome (PCOS), fertility complications (*e.g*., anovulation and endometriosis), pregnancy complications (*e.g*., pre-eclampsia, gestational diabetes, and recurrent pregnancy loss), and increased risk of breast and uterine cancer, among others.^4,6^ The prevalence of PCOS is estimated at 6%–10% in adults and 3%–11% in adolescents.^7,8^ PCOS and insulin resistance cause a hormonal imbalance resulting in follicle maturation arrest and defective ovulation.^6,9^ Obesity induces functional alterations in the hypothalamic–pituitary–ovarian axis, resulting in ovaries that are less responsive to gonadotropin stimulation.^10^ Taken together, fertility complications bring a significant number of women with obesity into the care of obstetrician-gynecologists (OBGYNs).

Currently, there are 4152 physicians certified in obesity medicine, of whom 179 are OBGYNs.^11^ OBGYNs are uniquely positioned for diagnosing and treating women with obesity, as they are often the only doctors a woman sees in her childbearing years. A survey of 935 OBGYNs revealed that an estimated 37% of their nonpregnant patients with private health insurance rely on them for primary care.^12^ In addition, OBGYNs are seeing progressively more patients with obesity, and hence are increasingly seeing associated complications (*e.g*., infertility, high-risk pregnancies, and gynecological cancers). However, the potential to treat obesity in the obstetrics and gynecology setting is not fully realized. A survey of 433 U.S. OBGYNs revealed that, while 90% had counseled patients for obesity often or most of the time, 52% had never prescribed antiobesity medications (AOMs), and only 27.2% routinely referred patients to other health professionals for weight management.^13^ This low rate of prescription and referral may be partially explained by pessimism about their ability to help, as 69% of OBGYNs thought they were unlikely/very unlikely to help their patients lose weight.^13^ Other barriers may include perceived lack of time, inadequate reimbursement,^14,15^ and possible challenges of accessing AOMs due to cost or prior authorization (PA) requirements. Therefore, it is important OBGYNs are aware of the treatment options for obesity and understand the benefits of AOMs.

Obesity treatment recommended by international and national guidelines comprises three tiers, starting with lifestyle intervention composed of dietary modification and increased physical activity, advancing to pharmacotherapy, and escalating to metabolic and bariatric surgery.^4,16,17^ Multiple pharmacological options can be considered for women with obesity. This review provides an overview of evidence supporting use of glucagon-like peptide-1 receptor agonists (GLP-1 RAs), the most recently approved AOM class and among the most efficacious ones.^18,19^ We discuss the role of OBGYNs in obesity care and suggest strategies for the initiation and maintenance of GLP-1 RA therapy, such as management of common side effects, to ensure that their patients achieve optimal therapeutic response.

## Risks of Obesity and Benefits of Weight Loss in Women

Contrary to the popular belief among patients that obesity is largely an esthetic issue (*i.e*., appearance and self-esteem),^20^ and the belief among physicians that obesity has a lower impact on health than other serious diseases,^21^ obesity is associated with the development or worsening of many other health conditions.^3^ These include some of the leading causes of death for women in the United States, including heart disease, stroke, some cancers, respiratory diseases, type 2 diabetes (T2D), and kidney disease.^22,23^ This puts obesity among the leading preventable causes of death, along with tobacco smoking.^24^

Obesity in women poses additional risks if they become pregnant. Along with an increased risk of death, pregnant women with obesity are at a higher risk of miscarriage, stillbirth, pre-eclampsia, gestational diabetes, and venous thromboembolism, compared with women of normal BMI (18.5 to <25 kg/m^2^).^25–27^ Obesity decreases the incidence of spontaneous labor and increases the risk of prolonged pregnancy and cesarean section, with associated risk of wound infections.^4,25,26,28,29^ In a systematic review and meta-analysis, risk of caesarean delivery in nulliparous women was 50% higher with overweight (BMI ≥25 to <30 kg/m^2^), double with class I obesity (mild obesity; BMI ≥30 to <35 kg/m^2^), and triple with class II/III obesity (moderate and severe obesity; BMI ≥35 to <40 and ≥40 kg/m^2^), compared with women of normal BMI.^30^

Health risks of maternal obesity for the fetus include iatrogenic prematurity, macrosomia, birth trauma (*e.g*., shoulder dystocia), and congenital anomalies.^4,25,26^ The risks of adverse maternal and neonatal outcomes relative to women of normal BMI increase with each BMI class, particularly in first-time mothers.^31,32^ Prepregnancy obesity has also been associated with up to 13% lower rates of breastfeeding initiation, and 20% decreased likelihood of breastfeeding at 6 months after birth.^33^ In a study of 185 healthy women in the United States, only 65% of women with obesity were exclusively breastfeeding 6 weeks after birth compared with 91% of women with normal weight.^34^ Long-term health risks for children born to mothers with high BMI include inheriting obesity (heritability estimated at 75% [47%–90%]), which may develop in the offspring's childhood or adulthood.^35,36^ Maternal obesity has also been linked with the offspring's development of coronary heart disease, T2D, stroke, asthma, and possibly poorer cognitive performance and renal health.^35–37^

**FIG. 1.**
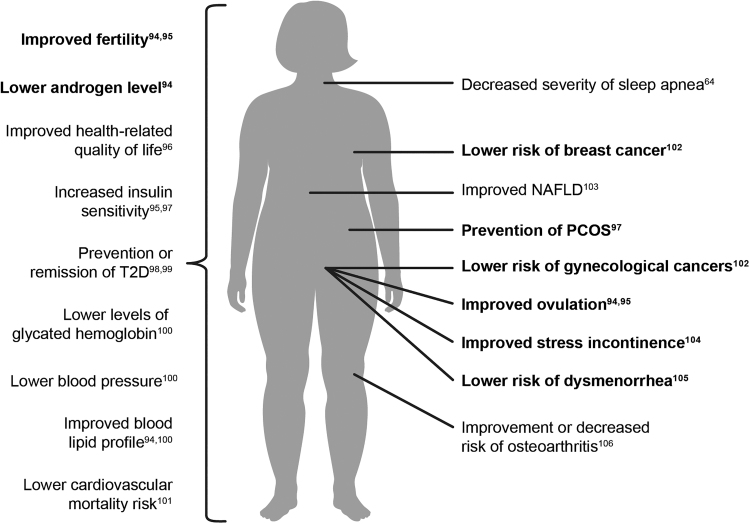
Benefits of weight loss in women.^94–106^ Bold text highlights effects specific for women's health. NAFLD, non-alcoholic fatty liver disease; PCOS, polycystic ovary syndrome; T2D, type 2 diabetes

International guidelines for obesity treatment recommend at least 5%–10% loss of initial weight, ideally reached within 6 months to achieve clinically meaningful benefits.^4,38^ In women with overweight/obesity and PCOS, it is recommended to lose at least 5%–15% of bodyweight to improve hyperandrogenism, infrequent periods, anovulation, insulin resistance, and hyperlipidemia, and at least 10% weight loss to increase the chances of conception and live birth.^16^ The long-term benefits of weight loss include prevention or improvement of other obesity-related complications, lower health care cost, and increase in quality of life (Fig. 1). For OBGYNs, successful weight loss in their patients may mean seeing increasingly healthy patients with ultimately less requirement for treatment for both obesity-related complications and infertility.

## Altering the Biology of Obesity with Pharmacotherapy

### Processes involved in development of obesity

Maintaining stable bodyweight requires a balance between energy intake and expenditure. However, achieving this state is not simple, as energy balance is regulated by complex feedback processes involving internal and external factors.^39^ Individuals trying to lose weight by making conscious changes in eating habits (*i.e*., the cognitive system) face the challenge of other (*i.e*., the hedonic and homeostatic) systems acting against the change.^39^ Energy restriction and subsequent weight loss stimulate powerful metabolic and hormonal compensatory mechanisms that promote weight regain and make long-term weight maintenance challenging.^40^ Pharmacological interventions can facilitate weight loss by targeting the homeostatic and hedonic regulation of food intake, resulting in decreased calorie absorption or reduced appetite.^41^

### Pharmacological options for treatment of obesity

There are currently four AOMs approved and marketed in the United States for chronic weight management. Orlistat has been in use since 1999, while phentermine/topiramate, naltrexone/bupropion, and liraglutide 3.0 mg were approved between 2012 and 2014.^42–45^ Modes of action and safety of the four AOMs are summarized in Table 1. Importantly, all of these AOMs are contraindicated in pregnancy,^42–45^ and so effective contraception must be used while on treatment.

**Table 1.** Safety Summary of the Four Currently Marketed Antiobesity Medications

	Orlistat	Phentermine/topiramate extended release	Naltrexone/bupropion	Liraglutide 3.0 mg
Mode of action	Inhibiting pancreatic lipase and thus reducing the amount of calories absorbed in the gut^43^	Combination of a sympathomimetic amine and an anticonvulsant, suppressing appetite and prolonging the feeling of satiety^45^	Combination of an opioid receptor antagonist and a dopamine/noradrenaline reuptake inhibitor working in synergy to influence appetite^44^	A long-acting analog of human GLP-1 (an incretin hormone) that increases glucose-dependent insulin secretion, decreases inappropriate glucagon secretion, suppresses appetite, and decreases energy intake^55^
Common side effects	Oily spotting, flatus with discharge, fecal urgency fatty/oily stool, oily evacuation, increased defecation, and fecal incontinence^43^	Paresthesia, dizziness, dysgeusia, insomnia, constipation, and dry mouth^45^	Nausea, constipation, headache, vomiting, dizziness, insomnia, dry mouth, and diarrhea^44^	Nausea, hypoglycemia, diarrhea, constipation, vomiting, headache, dyspepsia, fatigue, dizziness, abdominal pain, and increased lipase^42^
Patient withdrawal rate due to side effects	About 8%^107^	4.4%–19%^109,110^	19.5%–29.4%^112,113^	8.5%–12%^61–64^
Safety concerns	Decreased absorption of vitamins A, D, E, and K^107^ Potentially increased risk for colorectal cancer, as shown in animals—although not confirmed in humans^108^	Several neuropsychiatric warnings, including suicidal ideation, visual side effects, anxiety, and depression^45^ Increased heart rate^45^ Exposure to topiramate in the first trimester increases the risk of oral clefts in newborns^111^; ensure patient is not pregnant and test monthly, while on the drug^45^	Increased risk of seizures^114^ Cardiovascular safety trial terminated early and did not statistically confirm that the drug does not pose excess cardiovascular risk versus placebo^115^	Pancreatitis^42^ Higher risk of thyroid C-cell tumors in animals, but unknown risk in humans^42^

GLP-1, glucagon-like peptide-1.

Trials directly comparing the efficacy of different AOMs are limited. In the absence of such trials, relative efficacy of AOMs was compared by placebo-subtracted weight loss and indicated the highest efficacy for phentermine/topiramate, liraglutide, and naltrexone/bupropion.^18,19^ However, such comparisons should be interpreted with caution, as lifestyle interventions differed among the trials. Two studies involving a direct comparison reported significantly greater weight loss in patients receiving liraglutide 3.0 mg compared with those on orlistat (*p* < 0.001).^46,47^

Liraglutide 3.0 mg is the first AOM to mimic the action of a naturally occurring incretin hormone secreted by the gut. Liraglutide was originally developed as a treatment to improve glycemic control in patients with T2D (in 1.8 mg dose), but was found to also elicit reductions in bodyweight (trials with 39.1%–52.1% of women).^48–50^ In addition, in patients with T2D, liraglutide 1.8 mg was shown to have beneficial effects on cardiovascular and kidney outcomes (trial with 35.7% of women).^51,52^ Another randomized controlled trial (76.1% of women) investigated the optimal dose to maximize weight loss and identified that liraglutide 3.0 mg resulted in the greatest weight loss compared with liraglutide 1.2, 1.8, and 2.4 mg, with acceptable tolerability.^46^

### Mode of action of GLP-1 and GLP-1 RAs

GLP-1 is one of the peptide hormones involved in appetite regulation.^53^ It is primarily produced in intestinal L-cells and hindbrain neurons, and secreted into the circulation a few minutes following food ingestion.^53,54^ Endogenous GLP-1's role is to stimulate insulin and suppress glucagon secretion, promote glucose disposal, and reduce appetite and food intake.^53^ GLP-1's actions in the hypothalamus and digestive system result in increased feelings of satiety and reduced appetite, which facilitate weight loss through reduced energy intake.^53^

Liraglutide is a GLP-1 RA with a half-life of 13 hours following subcutaneous administration.^42^ This prolongation is achieved by reversible binding to endogenous serum albumin, facilitated by an added fatty acid side chain, and thus protection from degradation by dipeptidyl peptidase-4 or kidney filtration.^55^ Liraglutide 3.0 mg once daily is currently the only GLP-1 RA approved for chronic weight management.^42^ The dose of liraglutide for weight loss (3.0 mg) is higher than that for T2D (1.8 mg) because the 3.0 mg dose has resulted in greater weight loss than doses <3.0 mg, while doses >1.8 mg did not increase the glucose-lowering effect.^46,56,57^

Semaglutide is another GLP-1 RA, further modified to achieve a prolonged action allowing once-weekly dosing.^58^ It is currently approved for treatment of T2D only; however, trials investigating its efficacy and safety as an AOM are ongoing. Results from a phase 2 trial demonstrated even greater weight loss with semaglutide 0.2–0.4 mg daily versus liraglutide 3.0 mg,^59^ and phase 3 trials (the Semaglutide Treatment Effect in People with Obesity [STEP] program, NCT03548935) with semaglutide 2.4 mg weekly are ongoing. Promising weight-loss data have also been shown in a phase 2 trial with LY3298176 (tirzepatide), a dual glucose-dependent insulinotropic polypeptide and GLP-1 RA for the treatment of T2D^60^ currently being investigated in a phase 3 trial (SURMOUNT-1, NCT04184622).

## Results with Liraglutide 3.0 mg for Treatment of Obesity

### Efficacy of liraglutide 3.0 mg

The Satiety and Clinical Adiposity—Liraglutide Evidence (SCALE) trial program consisted of four key phase 3 trials investigating the effects of liraglutide 3.0 mg and placebo, each added to a lifestyle intervention of dietary modification and increased physical activity. The focuses of the SCALE trials were weight management and delayed onset of T2D (SCALE Obesity and Prediabetes),^61^ weight management in individuals with T2D (SCALE Diabetes),^62^ prevention of weight regain (SCALE Maintenance),^63^ and effect of liraglutide on sleep apnea (SCALE Sleep Apnea).^64^ Overall, 5358 adults (mean age 45–55 years) with obesity were enrolled in SCALE. Women comprised 48%–84% of patients in the first three trials,^61–63^ but only 28% in SCALE Sleep Apnea, as sleep apnea is more prevalent and severe among men.^64^ Subanalyses by sex in SCALE trials have not been published.

**FIG. 2.**
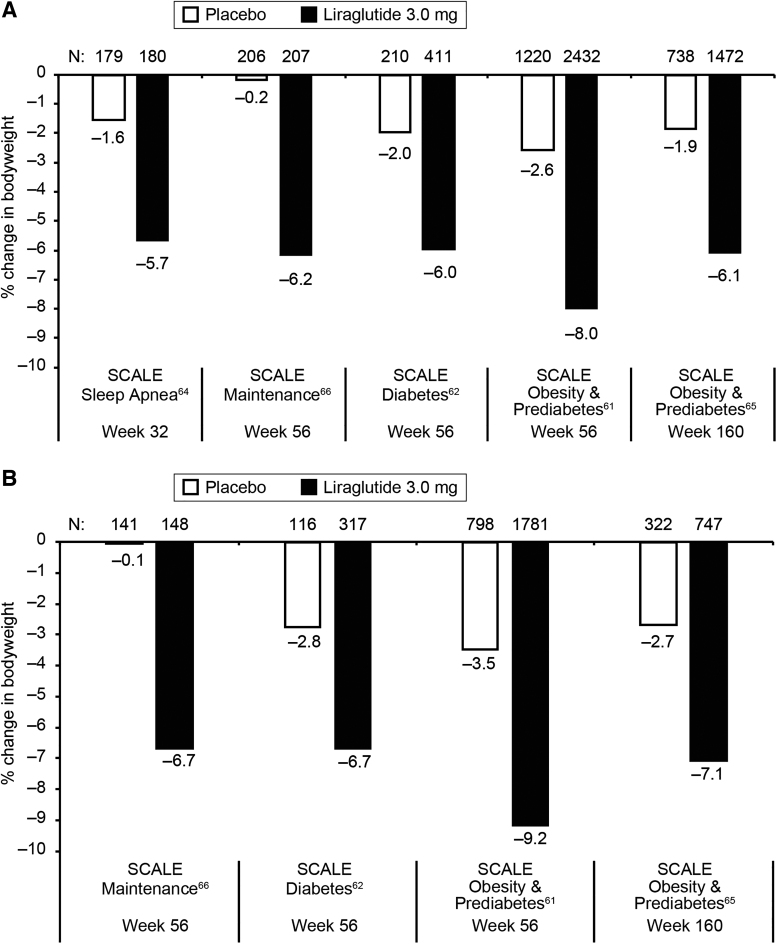
Efficacy of liraglutide 3.0 mg versus placebo across five SCALE trials.^61–65^ **(A)** Data from full analysis sets (all patients who underwent randomization and received at least one dose of a study drug and had at least one assessment after baseline). **(B)** Data from the “completer” populations (all patients in the full analysis sets with a valid, nonimputed measurement at trial end). Results from this population were not reported in the SCALE Sleep Apnea trial. SCALE, Satiety and Clinical Adiposity—Liraglutide Evidence.

In all four SCALE trials, significantly greater weight loss was achieved with liraglutide 3.0 mg compared with placebo (*p* < 0.001^61,62^ and *p* < 0.0001^63,64^). Individuals receiving liraglutide 3.0 mg lost on average 6.0%–8.0% of baseline bodyweight after 56 weeks (vs. 0.2%–2.6% with placebo),^61–63^ and 6.1% after 3 years (vs. 1.9% with placebo)^65^ (Fig. 2). In SCALE maintenance, the mean weight loss of 6.2% after 56 weeks was additional to a previous mean 6% weight loss achieved by a low-calorie diet (a daily deficit of 500 kcal) in 4–12 weeks.^63^ Maintenance of ≥5% weight loss was achieved by 81% of patients receiving liraglutide 3.0 mg for 1 year (vs. 49% with placebo; *p* < 0.0001).^63^ Furthermore, patients in SCALE receiving liraglutide 3.0 mg had significant improvements in cardiometabolic risk factors compared with placebo.^62,64,65^

The superiority of liraglutide 3.0 mg to placebo was also observed when given in addition to intensive behavioral therapy (IBT) in SCALE IBT (83% female, mean age 45–49 years). After 56 weeks, 142 patients receiving liraglutide 3.0 mg lost on average 7.5% of bodyweight, versus 4.0% loss in 140 patients with placebo (*p* = 0.0003).^66^ Weight loss of ≥5% was achieved by 62% and 39%, respectively (*p* = 0.0003).^66^ In a subanalysis of this trial, compared with nonadherence, complete adherence to dietary self-monitoring and physical activity was associated with estimated weight change of −7.2% (*p* < 0.0001) and −2.0% (*p* = 0.0009), respectively, and complete adherence to liraglutide 3.0 mg predicted an additional weight loss of −6.5% (*p* = 0.0005), while placebo did not have a significant effect on weight loss (1.9%, *p* = 0.33).^67^

Results from the recent SCALE insulin trial have demonstrated good efficacy of liraglutide 3.0 mg in patients with overweight/obesity and T2D (52% women, mean age 55.9–57.6 years) with concomitant basal insulin treatment and ≥2 antidiabetic drugs. After 56 weeks, mean change in bodyweight with liraglutide 3.0 mg was −6.5% versus −1.7% with placebo, without increasing hypoglycemic events or identifying new safety/tolerability issues.^68^

In women with overweight/obesity and PCOS, treatment with liraglutide 3.0 mg for 12–76 weeks also resulted in significant weight loss from baseline (*p* = 0.001^69^ and *p* < 0.0001^70^). Beneficial effects beyond weight loss were also reported in women with PCOS receiving liraglutide 1.8 mg, observed as improved ovarian function, higher menstrual regularity, and reduced androgen levels.^71^ In another study in women with PCOS and infertility, addition of liraglutide 1.2 mg to metformin for 12 weeks resulted in significantly higher pregnancy rate after *in vitro* fertilization compared with metformin alone (pregnancy rate per embryo transfer 85.7% vs. 28.6%, respectively; *p* = 0.03).^72^ As the weight loss between the combined therapy and metformin alone was not significantly different, it is possible that the mechanism of the beneficial effects of liraglutide on fertility stretches beyond weight reduction and improved insulin sensitivity, potentially involving the hypothalamic–pituitary axis.^72^

While liraglutide 3.0 mg is contraindicated in pregnancy, some data on pregnancy outcomes with exposure to liraglutide are available from clinical trials, observational studies, market research programs, literature, and spontaneously reported cases. Of 111 pregnancies with known fetal outcome, 53 (47.7%) resulted in live birth, of which 2 (1.8%) had a congenital anomaly.^73^ Fetal loss occurred in 38 (34.2%) pregnancies, due to spontaneous abortion (*n* = 32; 28.8%), ectopic pregnancy (*n* = 2; 1.8%), and stillbirth (*n* = 2; one with fetal defects).^73^ Finally, 20 (10%) pregnancies were terminated, of which 6 (5.4%) had reported fetal defects, and for 12 (10.8%), no reason was stated.^73^

The effect of liraglutide 3.0 mg in clinical practice was investigated in real-world studies. A retrospective observational study with 311 patients (83% female, mean age 50 years) reported significant weight loss with liraglutide 3.0 mg after ≥6 months of treatment (7.1%; *p* < 0.001).^74^ Weight loss of ≥5% and ≥10% was achieved by 64% and 35% of patients, respectively.^74^ In a study with 500 patients in a clinical setting (74% female, mean age 47–52 years), ≥5% weight loss was observed in 65% versus 27% of patients receiving liraglutide 3.0 mg or orlistat, respectively (*p* < 0.0001).^47^ However, it should be noted that both of these studies were performed at specialized weight management or obesity clinics, and so the outcomes at an OBGYN practice may differ.

Persistence with AOMs is crucial for weight loss and maintenance; it is important to study patients' long-term adherence to the medication in real-world settings. In a retrospective analysis of patient data, the persistence of 26,522 U.S. patients (79% female, mean age 47–49 years) with newly prescribed AOM was compared.^75^ The results indicated a significantly lower risk of treatment discontinuation with liraglutide 3.0 mg compared with other AOMs (*p* < 0.0001). After 1 year, 28.2% of patients were still taking liraglutide 3.0 mg, versus 10.9% on phentermine/topiramate and 9.2% on naltrexone/bupropion.^75^

### Safety and tolerability of liraglutide 3.0 mg

Gastrointestinal events are recognized as the most frequent side effects associated with GLP-1 RAs,^76^ which may be related to their effect on feelings of satiety and reduced gut motility.^77^

Across five double-blind, placebo-controlled trials assessing the safety of liraglutide 3.0 mg versus placebo, side effects occurring in >5% of patients (in a descending order) were nausea, hypoglycemia (in T2D), diarrhea, constipation, vomiting, headache, dyspepsia, fatigue, dizziness, abdominal pain, and increased lipase.^42^ Compared with placebo in patients with T2D, the risk of hypoglycemia was not increased with liraglutide 3.0 mg.^42,68^ Nausea occurred 2.8 times more frequently in patients receiving liraglutide versus those with placebo (39.3% vs. 13.8%, respectively). Diarrhea and constipation each occurred approximately twice as often in the liraglutide 3.0 mg group compared with placebo (20.9% vs. 9.9% and 19.4% vs. 8.5%, respectively), and vomiting was about four times more frequent (15.7% for liraglutide and 3.9% for placebo).^42^ Weight loss achieved with liraglutide 3.0 mg treatment is not a result of gastrointestinal adverse events, as there was no significant difference in weight loss between individuals with and without gastrointestinal events.^78^ Liraglutide 3.0 mg was associated with a slightly higher incidence of acute gallstone disease and mild-to-moderate pancreatitis.^42^ However, gallstone disease is associated with rapid weight loss regardless of how it is achieved.^79^

Liraglutide 3.0 mg is contraindicated in patients with personal or family history of thyroid C cell tumors due to a suspected risk based on preclinical data.^42^ However, in a phase 3 trial, no case of medullary thyroid carcinoma or C cell hyperplasia was observed in patients after 3 years of treatment with liraglutide 3.0 mg.^65^ Breast cancer was reported more often in patients receiving liraglutide 3.0 mg than those receiving placebo (0.7% and 0.2%, respectively); however, the cases were too few to determine whether liraglutide has an effect on pre-existing breast neoplasia.^42^

An increase in heart rate of 2–3 bpm was observed with liraglutide 3.0 mg^42^; however, in a large cardiovascular outcomes trial (Liraglutide Effect and Action in Diabetes: Evaluation of Cardiovascular Outcome Results [LEADER]), liraglutide 1.8 mg was associated with cardiovascular benefits.^52^ Regulatory authorities have accepted that findings from liraglutide 1.8 mg are relevant to the cardiovascular safety profile of liraglutide 3.0 mg, and this is reflected by the inclusion of LEADER data in the European and U.S. labels.^42,80^

## Assisting Patients with Initiating and Maintaining Therapy with Liraglutide 3.0 mg

It is important to recognize women who have overweight/obesity before pregnancy to allow time for counseling about bodyweight.^81^ OBGYNs should be encouraged to routinely discuss bodyweight with patients and play an active role in identifying weight-management solutions. Given the persisting stigma around obesity, physicians are encouraged to be mindful of their own implicit biases and choose a respectful and compassionate way to open the discussion about bodyweight with patients' permission.^82,83^ Open-ended questions about their physical abilities in everyday life, such as climbing stairs or getting up off the floor, can be asked. An overview of principles important for care of patients with overweight/obesity has been summarized and is updated annually by the Obesity Medicine Association.^17^ Further tips and guidelines for managing obesity in women have been summarized by Tauqeer et al.^84^

The weight loss achieved with health behavioral changes is usually 3%–5% of bodyweight, and it varies substantially based on the individual's biological and psychosocial factors.^83^ If weight loss or maintenance achieved with lifestyle intervention is not sufficient for the improvement of health and well-being, adjunctive pharmacotherapy should be considered.^83,84^

### Initiating liraglutide treatment

Liraglutide 3.0 mg is prescribed in addition to dietary modification and increased physical activity in adults with BMI ≥30 kg/m^2^ with or without comorbidities, or ≥27 kg/m^2^ with one or more weight-related comorbidities (*e.g*., hypertension, dyslipidemia, and T2D).^42^ It has recently been approved for use in patients with concomitant insulin treatment, with recommended dose reduction of antidiabetic drugs to reduce the risk of hypoglycemia.^42^ Contraindications include pregnancy, personal or family history of multiple endocrine neoplasia syndrome type 2 or medullary thyroid carcinoma (but not the more common and unrelated papillary thyroid cancer), and hypersensitivity to liraglutide or other product components. Liraglutide is not recommended for nursing women.^42^

Liraglutide 3.0 mg is currently only approved for use in adults.^42^ However, liraglutide 1.8 mg has recently been approved for T2D treatment in pediatric patients from 10 years,^85^ and recent evidence of using liraglutide 3.0 mg in 151 adolescents (12 to <18 years of age) has demonstrated a significant reduction in BMI standard deviation score versus placebo (*p* = 0.002).^86^ Given the growing prevalence of obesity in adolescents and the possible onset of PCOS at a young age,^87,88^ the potential future approval of liraglutide 3.0 mg for adolescent patients would represent an opportunity to initiate pharmacological weight management early, and reduce the risk of developing obesity and related complications in adulthood.

Prescription of liraglutide 3.0 mg can be given to young women over 18 years of age with overweight/obesity to prevent development of obesity-related complications, including infertility; women with overweight/obesity and infertility to increase chances of getting pregnant later and carrying pregnancy to term with fewer complications; women with overweight/obesity who have had children and are no longer nursing; or women with overweight/obesity who have not had children. If adolescent women with obesity present with suspected diabetes, OBGYNs should refer them to pediatric endocrinologists. Women of childbearing age should use effective contraception while on weight-loss therapy to prevent overlap between treatment and potential pregnancy, although there is no known teratogenicity. According to the stopping rule applicable in the United States, liraglutide 3.0 mg should be discontinued if the patient does not lose at least 4% of bodyweight after 16 weeks of treatment.^42^

The injections should be administered subcutaneously in the abdomen, thigh, or upper arm once a day, at any time of day (preferably at a similar time), and independent of meal times.^42^ It is recommended to start with a low dose of 0.6 mg, followed by weekly increments of 0.6 mg, until 3.0 mg is reached.^42^ We recommend that all staff (*e.g*., medical assistants and nurses) within a practice are familiar with the pen technique explained in the product instructions to ensure patients are trained and comfortable with liraglutide administration. The PA request form required by many insurance companies contains a few simple clinical questions that staff can answer from the patient's chart. However, many insurance companies do not cover AOMs, even with PA. Given the critical need to treat the obesity epidemic and the frontline position of physicians, completing PA forms could be a way to advocate for the patients and influence the healthcare coverage in the future.

Successfully treating obesity requires time, education, and accountability for patients. There are many programs available, both in the community and with the use of technology. Physicians may also consider training a staff member to be a point of contact for patients or become familiar with resources to provide to the patient.

### Managing common side effects

Gastrointestinal side effects are common, but usually mild to moderate and transient, mostly occurring in the first few weeks during dose escalation.^89^ Patients should be assured that the weight-loss effect is not related to the gastrointestinal side effects. There are several ways these side effects can be managed.

It is recommended that the daily dose is escalated slowly. If a dose increase is not well tolerated, the patient may temporarily de-escalate to previously tolerated dose and attempt the dose increase again in several days to 1 week, depending on the severity of the response. Dose timing can be changed according to patients' response to the treatment, for example, before bedtime. Patients should also be advised to adjust eating habits to help prevent side effects (*e.g*., eating small portions more often, chewing food well, limiting liquids at mealtimes, and avoiding strong smells and fatty foods) and may be advised to use ginger to relieve nausea.^90,91^

In more severe cases of nausea and vomiting, prescribing antiemetics may be considered. In a single-dose study with the GLP-1 RA exenatide, premedication with oral antiemetics metoclopramide and ondansetron hydrochloride significantly reduced the incidence of nausea and vomiting.^92^ However, care should be taken, as antiemetics may cause drowsiness or worsen constipation. The option of prescribing antiemetics should not reduce the extent of patient education before treatment initiation and support provided during the dose-escalation phase.

### Helping patients with long-term treatment adherence

Persisting with AOMs is crucial for achieving and maintaining weight loss while the patient continues with dietary modification and increased physical activity. Patients should be reminded of the many health benefits of losing weight, including prevention, improvement, or even remission of chronic diseases, increased fertility, and better pregnancy outcomes. Patients should be encouraged to achieve and maintain ≥5% to 10% weight loss. It is strongly recommended that patients set their nutrition and exercise goals and self-monitor their progress, including weighing regularly, at least once a week. Any goal-setting should follow the SMART method (Specific, Measurable, Attainable, Realistic, and Time-bound) to facilitate progress. OBGYNs are encouraged to maintain frequent follow-up with patients (*e.g*., once a month at the beginning and every ≤3 months thereafter) and review their nutrition, exercise habits, sleep habits, current stressors, and upcoming challenges (*e.g*., holidays), and set goals for the next follow-up. Alternatively, other members of staff at the practice could assist with maintaining frequent follow-up with the patients between visits with the OBGYN.

According to a meta-analysis on adherence to weight-loss interventions, patients are more likely to adhere to those that are supervised, offer social support, and focus on both dietary and exercise goals (as opposed to exercise alone).^93^ Given that factors such as older age, male gender, or hyperlipidemia were associated with higher adherence to AOMs, younger female patients with normal blood-lipid profiles may, on average, require more effort to stay motivated.^75^ OBGYNs may use motivational interviewing to enhance patients' inner motivation and promote behavioral change.^17^ Social support can be driven by family, friends, peers, providers, or social support contracts.^66^ Dietary goals could involve portion sizes, calorie intake, quantity of fruits/vegetables, a time cutoff for the last meal, or frequency of cooking own meals. Physical activity goals may include caloric expenditure, time dedicated to a physical activity, number of steps, or time to cover a distance. Many of these goals and support systems can be facilitated with online tools and application software designed to track progress and offer group support.

## Conclusions

Obesity in women imposes an increasing healthcare burden that extends to long-term health risks for their offspring. In this review, we aimed to inform the OBGYNs of the options for and benefits of antiobesity treatments and offer encouragement to play an active role in obesity care. Despite the time restriction, OBGYNs remain one of the limited points of healthcare for women of reproductive age, and thus, a continued education regarding options of the treatment of obesity is extremely important. OBGYNs can play an essential role in helping patients start and persist with AOM treatment, and can offer practical help with achieving weight loss and managing side effects. Specifically, we have discussed how OBGYNs can support their patients treated with GLP-1 RAs. Treating obesity in OBGYN patients could lead to fewer obesity complications, higher chance of future pregnancy, and fewer health risks for their children.
